# Interferon-β decreases LPS-induced neutrophil recruitment to cardiac fibroblasts

**DOI:** 10.3389/fcell.2023.1122408

**Published:** 2023-09-20

**Authors:** Renatto Anfossi, Raúl Vivar, Pedro Ayala, Fabiola González-Herrera, Claudio Espinoza-Pérez, José Miguel Osorio, Mauricio Román-Torres, Samir Bolívar, Guillermo Díaz-Araya

**Affiliations:** ^1^ Unidad de Farmacia, Hospital Regional del Libertador Bernardo O’Higgins, Rancagua, Chile; ^2^ Departamento de Química Farmacológica y Toxicológica, Facultad de Ciencias Químicas y Farmacéuticas, Universidad de Chile, Santiago, Chile; ^3^ Instituto de Farmacología, Facultad de Medicina, Universidad de Chile, Santiago, Chile; ^4^ Facultad de Medicina, Pontifica Universidad Católica de Chile, Santiago de Chile, Chile; ^5^ Facultad de Química y Farmacia, Universidad del Atlántico, Barranquilla, Colombia; ^6^ Advanced Center for Chronic Diseases (ACCDiS), Facultad de Ciencias Químicas y Farmacéuticas, Universidad de Chile, Santiago, Chile

**Keywords:** Interleukin-8, neutrophils, cardiac fibroblast, metalloprotease, TLR4

## Abstract

**Introduction:** Cardiac fibroblasts (CF) are crucial cells in damaged heart tissues, expressing TLR4, IFN-receptor and responding to lipopolysaccharide (LPS) and interferon-β (IFN-β) respectively. While CF interact with immune cells; however, their relationship with neutrophils remains understudied. Additionally, theimpact of LPS and IFN-β on CF-neutrophil interaction is poorly understood.

**Methods:** Isolated CF from adult rats were treated with LPS, with or without IFN-β. This study examined IL-8 secretion, ICAM-1 and VCAM-1 expression, and neutrophil recruitment, as well as their effects on MMPs activity.

**Results:** LPS triggered increased IL-8 expression and secretion, along with elevated ICAM-1 and VCAM-1 expression, all of which were blocked by TAK-242. Pre-treatment with IFN-β countered these LPS effects. LPS treated CF showed higher neutrophil recruitment (migration and adhesion) compared to unstimulated CF, an effect prevented by IFN-β. Ruxolitinib blocked these IFN-β anti-inflammatory effects, implicating JAK signaling. Analysis of culture medium zymograms from CF alone, and CF-neutrophils interaction, revealed that MMP2 was mainly originated from CF, while MMP9 could come from neutrophils. LPS and IFN-β boosted MMP2 secretion by CF. MMP9 activity in CF was low, and LPS or IFN-β had no significant impact. Pre-treating CF with LPS, IFN-β, or both before co-culture with neutrophils increased MMP2. Neutrophil co-culture increased MMP9 activity, with IFN-β pre-treatment reducing MMP9 compared to unstimulated CF.

**Conclusion:** In CF, LPS induces the secretion of IL-8 favoring neutrophils recruitment and these effects were blocked by IFN-. The results highlight that CF-neutrophil interaction appears to influence the extracellular matrix through MMPs activity modulation.

## 1 Introduction

Cardiac fibroblasts (CF) are mesenchymal cells, traditionally visualized as the cells that produce and remodel the extracellular matrix (ECM) (Davis and Molkentin, 2014). In addition, CF are recognized as sentinel cells (Dia et al., 2015), because they express a wide variety of receptors, through which they respond to cytokines, chemokines, and growth factors, also releasing cytokines and growth factors that directly impact the surrounding cells and orchestrate the inflammatory infiltrate (Van Linthout et al., 2014). Among these receptors, the Toll Like Receptors (TLR) stand out, through which the CF have been shown to participate in the inflammatory response (Lorne et al., 2010). TLRs respond to pathogen-associated molecular patterns (PAMPs) and damage-associated molecular patterns (DAMPs). The presence of TLR4 has been demonstrated in various cell types and specifically in CF (Boza et al., 2016). Lipopolysaccharide is an active agonist of TLR4, leading to the activation of nuclear transcription factor kB (NF-kB), and interferon regulatory factor 3 (IRF3), which move to the nucleus to induce the transcription of specific genes, including proinflammatory cytokines and other immunoregulatory molecules (Arumugam et al., 2009; Mesquita et al., 2014). CF secrete IL-8 (Skioldebrand et al., 2017; Li et al., 2021), one of the major chemokines mediating inflammation, which contributes to the chemoattraction and activation of neutrophils to sites of tissue damage during inflammatory processes (Harada et al., 1994). The production of IL-8 is stimulated by inflammatory molecules, where LPS, IL-1β, and TNF-α stand out (Takahashi et al., 2001; Kato and Kitagawa, 2006). In rats this chemokine is known as Growth-regulated gene product/cytokine-induced neutrophil chemoattractant (GRO/CINC)-1 with structural and functional homology to human IL-8. CF also express the cell adhesion molecules ICAM and VCAM, which are involved in intercellular communication and are fundamental in the adhesion of inflammatory cells to sites of injury. In addition, CF actively participate in the immune response after tissue damage by secreting MMPs, which remodel extracellular matrix to allow immune cells infiltration (Turner and Porter, 2012).

Neutrophils are highly phagocytic cells that constitute the first line of defense of the innate immune system. It has been shown that the rapid degranulation of neutrophils is an important step in the initiation of inflammation, which results in the release of cytokines, the subsequent recruitment of inflammatory monocytes and the initiation of a secondary inflammatory response. Stimulation of neutrophils by IL-8 facilitates their adhesion to endothelial cells through the expression of cell adhesion molecules and integrins; it favors their ability to migrate, changes their shape, and regulates the exocytosis of storage proteins (Wright et al., 2010). Primary role of neutrophils is to eliminate pathogens by phagocytosis through the release of enzymes and granule proteins, and by the production of a range of reactive oxygen species (Segal, 2005). The adhesion of neutrophils to different cell types, such as myocytes, endothelial cells, and CF have been widely described which is dependent on ICAM and VCAM expression on these cells (Smith et al., 1991; Ban et al., 1994; Ikeda et al., 1994; Couture et al., 2009).

Interferon-β (IFN-β) is a cytokine that elicits a wide range of biological responses, often in a cell-type-specific manner (Noppert et al., 2007; Ivashkiv and Donlin, 2014). CF express and release IFN-β and its receptor (Zurney et al., 2007). IFN-β after binding to its receptor activates the intracellular signaling pathway Janus kinase (JAK)/Signal Transducer and Activator of Transcription (STAT). Once activated, STAT proteins form dimers and associate with IRF3, and then bind to IFN-β-mediated response elements in the cell nucleus (Darnell et al., 1994; Ihle, 1995; Chen et al., 2004). The participation of IFN-β is well-known in cells of the immune system (Lopez de Padilla and Niewold, 2016). On the contrary, there is little evidence regarding its function in cells other than those associated with the immune system, such as CF, to which a determining role in the inflammatory response has been attributed in recent years (Bolivar et al., 2018). Our previous studies have demonstrated the participation of IFN-β in the inflammatory response mediated by CF (Bolivar et al., 2018).

To date, there is evidence that has shown the release of IL-8 by CF; however, whether IL-8 secreted by CF is responsible for neutrophils recruitment, and the consequences of CF-neutrophil interaction are not fully known. Thus, the main aim of this study were to study in CF the IL-8 secretion by TLR4 activation, as well as, the recruitment of neutrophils and the consequences between CF and neutrophils interaction.

## 2 Materials and methods

### 2.1 Materials

Anti-GAPDH and anti-rabbit were purchased from Sigma-Aldrich Co. (St. Louis, MO, United States). DMEM/F12 culture medium, fetal bovine serum (FBS), and trypsin-EDTA were purchased from Gibco BRL (Carlsbad, United States). Anti-ICAM and anti-VCAM were purchased from Santa Cruz Biotechnology (Dallas, United States). Anti-RP-1 was obtained from BD Biosciences (San Jose, CA, United States). Histopaque-1083 and histopaque-1119 were purchased from Sigma-Aldrich Co. (St. Louis, MO, United States). Organic and inorganic compounds, salts, acids, and solvents were purchased from Merck (Darmstadt, Germany). LPS (Ultra pure lipopolysscharide from *E Coli* 0111:B4 strain-TLR4 ligand) Cat. code TLRL pelps, was purchased from Invivogen (San Diego, Ca, United States), TAK-242, and Collagenase type II were purchased from Invivogen (San Diego, United States). Sterile plastic material for obtaining and culturing cells was purchased from Falcon (Ballerica, Ca, United States). Xylazine 2% was purchased from Laboratorios Centrovet (Santiago, Chile). Ketamine 100 mg/ml was purchased from Richmond Vet Pharma (Bs. As., Argentina). Interferon-beta (IFN-β) was purchased from InterferonSourse (NJ, United States). MACS^®^ culture medium for neutrophil isolation was purchased to Miltenyi Biotec (Teterow, Germany).

### 2.2 Cardiac fibroblasts culture

Adult Sprague-Dawley rats (200–300 g) of 8 weeks of age were used to obtain primary cultures of CF. The animals were acquired from the Bioterio of the Faculty of Chemical and Pharmaceutical Sciences, University of Chile, in compliance with the ethical norms referring to the use of animals, with the approval of the Ethics Committee of the Faculty of Chemical and Pharmaceutical Sciences. For CF isolation, we used the procedure described previously (Lopez de Padilla and Niewold, 2016), with some modifications. Briefly, an adult rat was anesthetized intraperitoneally with a ketamine-xylazine mixture (66 and 1.6 mg/kg respectively). The heart was then excised, and the atria were separated from the ventricles. The latter were broken up mechanically into small pieces by adding 30 mL of a type II collagenase solution (1 mg/mL) and placing for digestive agitation at 37°C for 1.5 h. The digestion product was centrifuged at 500 rpm for 2 min at room temperature. The pellet resulting from this first centrifugation, rich in cardiomyocytes, was discarded and the supernatant, enriched in CF, was subjected to a second centrifugation at 1,000 rpm for 10 min at the same temperature. The obtained pellet was resuspended in 4 ml of DMEM-F12 medium supplemented with 10% FBS. Finally, the CF were seeded in 100-mm culture plates and incubated in a 5% CO2 and 37°C environment. After 24 h, the culture medium was changed to remove cell debris, erythrocytes, and non-adhered cells. The CF were incubated again under the same conditions mentioned above until 90%–100% confluency. They were then expanded to passage 1, after washing the cultures and treating them with trypsin-EDTA.

### 2.3 Cell passage

To carry out all the experiments, the cells were maintained at passage 0 (p0) in DMEM/F12 medium supplemented with 10% FBS, until reaching 100% confluence. Subsequently, the CF were released with 0.1% trypsin in sterile 1X PBS and counted using the trypan blue exclusion method. The cells were then seeded in 35 mm plastic cell culture plates at a concentration of 150,000 cells per plate for the determination of the ICAM and VCAM adhesion proteins, or in 60 mm plates at a concentration of 300,000 cells for the determination of the IL-8 protein, in DMEM/F-12 medium supplemented with 10% FBS (passage 1). Once adhered to the plate, the cells were washed with sterile 1X PBS and kept in DMEM/F-12 medium for 24 h until their subsequent treatment with the different stimuli to perform the experiments.

### 2.4 Rat cardiac fibroblasts treatments

Confluent first-passage adult rat cardiac fibroblasts were cultured as described before. Fibroblasts were treated for 24 h with LPS (1 μg/mL), and TAK-242 (4 μM, TLR4 antagonist) was added 30 min before LPS. In CF treated with IFN-β, the Recombinant human IFN-β (500 IU/mL) was added 1 h before LPS; while, Ruxolitinib (500 nM, a JAK inhibitor) was added 30 min before IFN-β. Saline solution was used as control for LPS. DMSO was used as vehicle for TAK-242 or ruxolitinib.

For experiments testing CF-neutrophils co-culture, fibroblasts were pretreated as following for 24 h (LPS, IFN-β, Ruxolitinib, IFN-β + LPS, Ruxolitinib + IFN-β, Ruxolitinib + IFN-β + LPS), and non-stimulated neutrophils were added for 2 h for adhesion assays. For MMPs assays CF were treated as following for 24 h (LPS, IFN-β, Ruxolitinib, IFN-β + LPS, Ruxolitinib + IFN-b + LPS). Then neutrophils were added, and the co-culture between CF and neutrophils was incubated by additional 48 h.

For experiments testing IL-8, ICAM1 and VCAM1 mRNA expression in CF, fibroblasts were stimulated with LPS (1 μg/mL) for 4, 8 and 24 h. Whereas for experiments testing TLR4 participation on LPS effects, fibroblasts were pretreated with TAK-242 (4 μM, TLR4 antagonist) for 30 min and then were stimulated with LPS (1 μg/mL) for 8 h. Then mRNA was obtained by TRIZOL method.

### 2.5 Western blot (WB)

The protein extracts obtained after CF cell lysis were quantified using the Bradford method and, subsequently, they were subjected to electrophoresis in 10% (ICAM and VCAM) acrylamide/bisacrylamide gels for the separation and resolution of proteins according to their mass. The concentrating gel (stacking) was 5%. Electrophoresis was carried out at a constant voltage of 100 V, keeping the amperage constant (350 mAmper). Then, the proteins were transferred for 90 min to nitrocellulose membranes with a 0.2 µm pore. The membranes were blocked with 5% non-fat milk in TBS-Tween. Nitrocellulose membranes were incubated with anti-ICAM1 (1:200), anti-VCAM1 (1:200) and anti-GAPDH (1:10,000) primary antibodies overnight, at 4°C, with gentle shaking. Then membranes were washed with TBS-Tween and incubated with an anti-rabbit secondary antibody (1:5,000). An ECL chemoluminescent method was used for the detection of enzyme activity.

### 2.6 IL-8 secretion assay

IL-8 secretion levels were measured from the CF culture medium grown in 60 mm dishes, using a commercial ELISA Kit (IL-8 ELISA Kit, MyBioSource, San Diego, CA, United States).

### 2.7 Neutrophils isolation from bone marrow

Rats were anesthetized intraperitoneally with a ketamine-xylazine mixture (66 and 1.6 mg/kg respectively). The thoracic cap was removed by bilateral thoracotomy and the heart was excised. The femur and tibia were extracted from both extremities and their surrounding tissue was removed. We then proceeded to the excision of the epiphysis at the level of the metaphysis of both bone types. The bone marrow was then removed by influx of DMEM/F12 medium via a 25G wide syringe onto a 100 mm Petri dish. Next, the suspension of cells rich in leukocytes and erythrocytes was transferred to 1.5 ml Eppendorf tubes and centrifuged at 300 g for 10 min at room temperature. The erythrocytes present in the suspension were then lysed with 0.83% NH4Cl for 7 min. Subsequently, they were washed twice with MACS^®^ medium and subjected to incubation with anti-RP-1 antibody at the ratio of 0.5 µL of anti-RP-1 per 10^6^ cells in 100 µL of total volume for 30 min, avoiding light. Next, the leukocyte suspension was washed twice with MACS^®^ buffer medium, and the analysis was carried out using Cell Sorting (Beckson-Dickinson FACS flow and sorting cytometer).

### 2.8 Cell Sorting

Cell populations were identified and separated by Cell Sorting (Becton-Dickinson FACS flow and sorting cytometer) using the anti-RP-1 antibody specific for neutrophils (Supplementary Figure S1). The total cell population can be appreciated by relating their cellular complexity (SSC) to cell size (FSC); phycoerythrin fluorescence (PE) RP-1 is displayed using a pseudocolor and dot plot format (Supplementary Figures S1A–D). In Supplementary Figure S1B and Supplementary Figure S1D, the population of neutrophils detected by the anti-RP-1 antibody can be distinguished, which is associated with the characteristic complexity of this cell type. In this case, it corresponds to 20% of the total cell population analyzed (Supplementary Figures S1A, C). To further clarify the identification of the neutrophil population in relation to the total cell population analyzed, this relationship is represented in the diagram in Supplementary Figure S1E (which represent the fusion of Supplementary Figures S1C, D), with its respective histogram analysis (S1F). Subsequently, in figures S1G and S1H, the results of the analysis of the total cell population after undergoing separation by Cell Sorting are shown, specifically displaying only the neutrophil population. The Post Sort analysis revealed that the neutrophil population was separated with 95% viability and 98% purity. Data analysis was done using the FlowJo software for reading histograms, according to the protocols indicated by the manufacturer.

### 2.9 Neutrophils migration assays

*In vitro* cell migration assays were performed using a Corning Transwell^®^ plate (Corning CLS3422, polycarbonate membrane cell culture inserts 6.5 mm Transwell with 8.0 μm pore polycarbonate membrane insert). Rat neutrophils (5x10^6^) were resuspended in 1 ml of DMEM/F12 maintenance medium and labeled with Calcein AM^®^ fluorescent probe (5 µM) for 30 min at 37°C. Labeled cells were washed twice with DMEM/F12 and 100 µL (5 × 10^5^ neutrophils) were added to the top compartment of the Transwell plate. In the lower compartment, 500 µL of conditioned culture medium were added, obtained from CF previously treated with LPS for 24 h in the presence/absence of IFN-β with/without ruxolitinib. Cells from the upper compartment were allowed to migrate to the lower compartment within 3 h at 37°C (5% CO_2_/95% air). Once the time elapsed, the fluorescence emitted by the neutrophils that had migrated to the lower compartment of the Transwell plate was measured, using a fluorescence spectrometer (excitation 470 and emission 517), equipped with a plate reader (Synergy 2 Multi-Mode reader, BioTek, United States). As a control of the technique, DMEM/F12 was used in the lower compartment, while DMEM/F12 + 10% FBS was used as a positive migration control. In some experiments, the conditioned medium obtained from the CF was preincubated for 24 h with the purified rat anti-IL-8 antibody (6 μg/ml) or IgG (used as control) prior to the migration assay.

### 2.10 Neutrophil adhesion assay on cardiac fibroblast monolayer

Cell adhesion was analyzed by Cell Sorting (Becton-Dickinson FACS flow and sorting cytometer). Leukocytes obtained from rat bone marrow were resuspended in DMEM/F12 medium at 1.5x10^6^ cells/100 µL and added to 35 mm dishes on a confluent monolayer of CF (15x10^4^) previously treated with LPS, IFN-β, IFN-β +LPS or Ruxolitinib + IFN-β + LPS. Following a 2 h incubation period at 37°C, the cell plates were washed with serum free medium and the CF with adherent neutrophils were detached using 0.1% trypsin in sterile 1X PBS. Then, the suspension of CF and neutrophils was washed with MACS^®^ medium at 300 g for 5 min at room temperature and incubated with anti-RP-1 antibody at the ratio of 0.5 µL of anti-RP-1 per 10^6^ cells in 100 µL of full volume for 30 min, avoiding light. Next, the leukocyte suspension was washed twice with MACS^®^ medium, and the analysis was carried out using Cell Sorting (Becton-Dickinson FACS flow and sorting cytometer). The percentage of neutrophil adhesion was calculated as follows: 100 x (number of RP-1+ attached cells/total number of RP-1+ added cells).

### 2.11 Co-culture experiments

In this study, we performed co-cultures of treated CF and Neutrophils (1 x 10^6^). Depending on the experiment and before co-incubation with the neutrophils, the CF were previously treated with IFN-β in the presence or absence of ruxolitinib (a JAK inhibitor), and then were treated with LPS for 24 h. In adhesion assays co-culture was performed by 2 h. In MMPs activity, the co-culture was performed by 48 h, then cultured media was collected and gelatinase activity was measured.

### 2.12 Gelatin zymogram metalloprotease activity assay

Metalloproteases 2 (MMP-2) and MMP-9 activities were assessed by *in vitro* gel zymography. Metalloproteinases produced gelatin lysis (semi quantified by reverse-image densitometry), and zymogen activation was suggested by doublet band formation. The CF were treated with IFN-β (500 UI/mL) for 1.5 h before LPS (1 μg/mL) for 24 h, and ruxolitinib (500 nM) was added 30 min before IFN-β. After this time, neutrophils (1x10^6^) were added and allow interacting for 48 h. The culture medium was collected, centrifuged and the supernatant was analyzed for gelatin activity. Conditioned medium was collected, and protein content was determined using the Bradford assay (Bio-Rad protein stain reagent) against a bovine serum albumin standard. Measurement of MMP activity per 10 μg of protein was obtained by gel zymography with 10% gelatin (Bio-Rad) as substrate. Cell culture medium was loaded onto 8% SDS-PAGE gels containing 1 mg/ml gelatin under non-reducing conditions and run at 120 V for 90 min, along with molecular weight standards. Gels were then washed four times in zymogram renaturation buffer (2.5% Triton X-100) and incubated overnight in zymogram running buffer (50 mM Tris-HCl, pH 7.5, 200 mM NaCl, 5 mM CaCl_2_, 0.02% NaN_3_). The gels were then stained with Coomassie Blue R-250 and then washed with 40% (v/v) methanol, 10% (v/v) acetic acid, and 50% water. Clear bands of MMP activity were visible against the blue background.

### 2.13 Quantitative real-time PCR

Total RNA was isolated using the TRIzol reagent (Invitrogen) and purified using the PureLink RNA Mini Kit (Thermo Fisher). RNA quantity and the 260/280 ratio were determined using a NanoDrop 2000 (Thermo Fisher). All RNA samples exhibited a 260/280 ratio within the range of 1.8–2.1. The RNA samples were stored at -80°C until cDNA synthesis was performed. cDNA synthesis was carried out using 1 μg of total RNA via reverse transcription, employing M-MLV Reverse Transcriptase (Invitrogen), random primers (Invitrogen), dNTPs (Promega), and RNAse OUT (Thermofisher). The program proceeded as follows: 65°C (5 min), 37°C (2 min), 25°C (10 min), 37°C (50 min), and 70°C (15 min). The resulting samples were stored at -20°C until qPCR was performed. Amplification was executed on an ABI Prism 7300 sequence detector (Applied Biosystems). Each reaction mixture comprised 250 nM of each primer, 10 ng of cDNA, 10 μL of SYBR Green from the SensiFAST™ SYBR^®^ Hi-ROX Kit (Bioline), 0,3 μL of ROX (diluted 1:50), and H20, resulting in a total volume of 20 μL. The cycling program included an initial denaturation step at 95°C for 10 min, followed by 40 amplification cycles at 95°C (30 s), 60°C (30 s), and 72°C (30 s). The final step involved a dissociation stage ranging from 60°C to 95°C (100 s). Relative quantification analysis was carried out, expressing the results as an RQ value determined through the comparative control (ΔΔCq) method RQ = 2^(sample ΔCq–control ΔCq). Three independent experiments were conducted, and each sample was measured in duplicate. As a negative control, nucleases-free water was employed instead of cDNA (NTC), yielding a Cq value of 0 (undetectable) for all analyzed genes (Table 1).

**TABLE 1:** qPCR primers sequence.

Gene	Forward primer (5′-3′)	Reverse primer (5′-3′)	NCBI reference sequence	Product length
*Il-8 (Cxcl1)*	CAT​TAA​TAT​TTA​ACG​ATG​TGG​ATG​CGT​TTC​A	GCC​TAC​CAT​CTT​TAA​ACT​GCA​CAA​T	NM_030845.2	76 bp
*Icam1*	GCC​TGG​GGT​TGG​AGA​CTA​AC	CAC​TCG​CTC​TGG​GAA​CGA​ATA	NM_012967.1	176 bp
*Vcam1*	TAC​AAG​TCT​ACA​CCT​CCC​CC	GCA​ATT​AAG​GTG​AGG​GTG​GC	NM_012889.2	190 bp
*Gapdh*	TGC​ACC​ACC​AAC​TGC​TTA​GC	GGC​ATG​GAC​TGT​GGT​CAT​GAG	NM_017008.4	87 bp

For the analysis of primer pair efficiency, a serial dilution of the sample pool was performed at ratios of 1, 1:10, 1:100, 1:1,000, and 1:10,000. The efficiency percentage (E%) was determined using the following formula: E% = (10^(-1/m) - 1) * 100, where “m” represents the slope of the LOG (Cq) vs. Cq graph. All *r* ^2^ values were > 0.99 and all amplification efficiency between 90% and 103% (Supplementary Figure S2). The specificity of the primers has been confirmed by melting curve analysis (Supplementary Figure S3).

### 2.14 Results expression and statistical analysis

The results are shown as mean ± SEM of at least three independent experiments (n ≥ 3). The data obtained were analyzed by ANOVA and the Tukey test, which allowed determining the statistical significance of the results, considering the difference between two groups significant when their *p*-value was less than 0.05.

## 3 Results

### 3.1 Determination of IL-8 protein levels in cardiac fibroblasts

**FIGURE 1:**
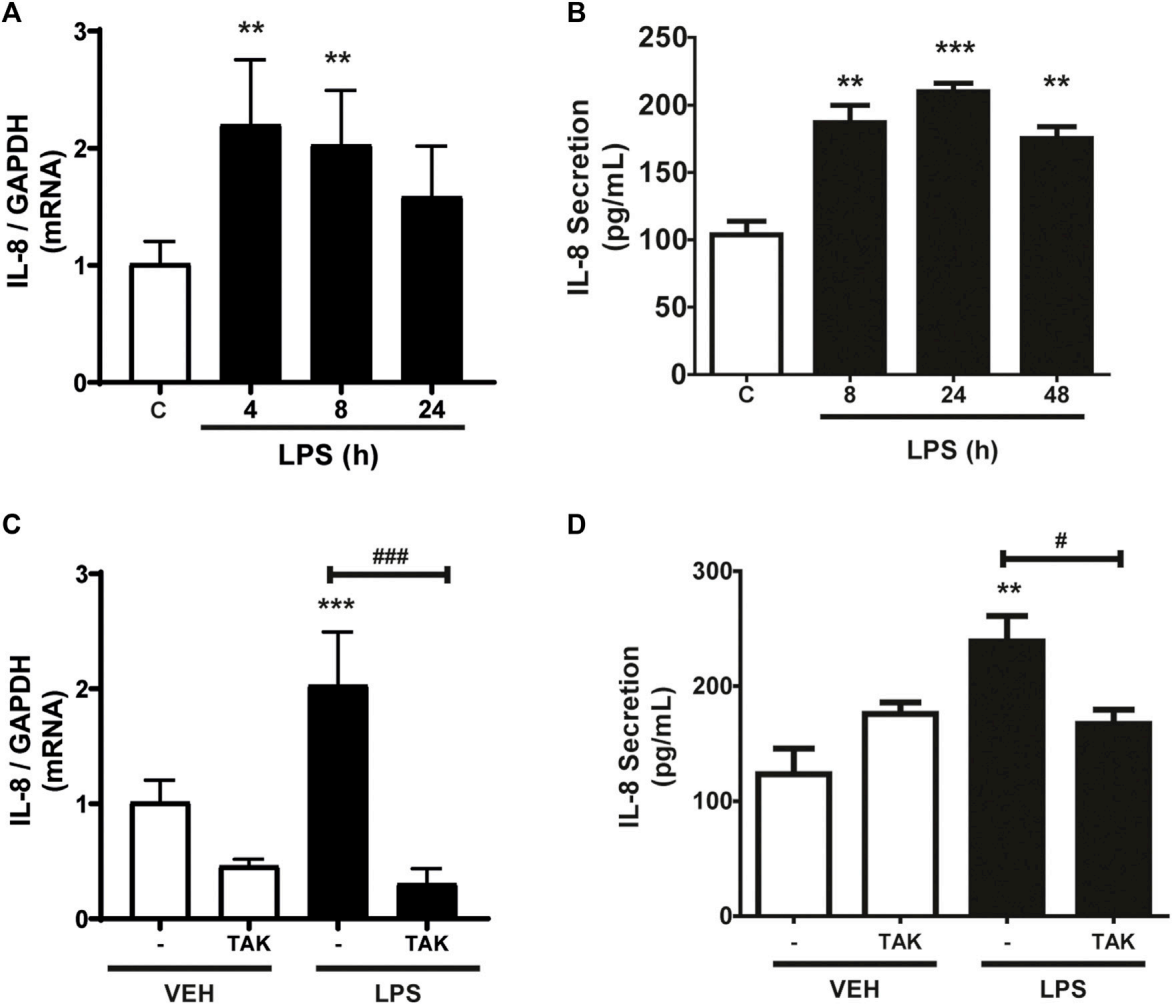
LPS induces the expression and secretion of IL-8 in cardiac fibroblasts (CF). **(A)** CF were stimulated with LPS (1 μg/ml) for 4, 8, and 24 h mRNA of IL-8 was analyzed by RT-qPCR. **(B)** CF were stimulated with LPS (1 μg/ml) at 8, 24 or 48 h. Secreted IL-8 levels were quantified using an ELISA kit. **(C)** CF were pre-stimulated with the TLR4 inhibitor TAK-242 (4 uM) and subsequently stimulated with LPS (1 μg/ml) for 8 h. IL-8 mRNA was determined with an RT-qPCR. **(D)** IL-8 secretion via TLR4 in CF. CF were pre-stimulated with the TLR4 inhibitor TAK-242 (4 uM) and subsequently stimulated with LPS (1 μg/ml) for 24 h. IL-8 secretion was determined with an ELISA kit. VEH: DMSO (vehicle for TAK-242). ***p < 0.001; **p < 0.01; *p < 0.05 vs. control; #*p* < 0.05 vs. LPS (-). Results are expressed as mean ± SEM. (*n* = 3).

As shown in Figure 1A, CF express IL-8, whereas incubation with LPS (1 μg/ml) increased IL-8 mRNA expression levels. At 4 h, the increase was statistically significant, but then returned to basal expression levels 24 h after incubation with LPS. We then studied the secretion of IL-8 into the extracellular medium. Figure 1B shows the IL-8 release profile from CF when stimulated for 8, 24, or 48 h with LPS (1 μg/ml) measured by ELISA. The results show that there was an increase in the secretion of IL-8 after 8 h of stimulation, which remained stable until 48 h. From these results, we could infer that IL-8 has a period of synthesis and a subsequent period of secretion. Then we proceeded to determine if the expression and secretion of IL-8 induced by LPS was dependent on the activation of TLR4. CF were pre-stimulated with the TLR4 inhibitor, TAK-242 (4 μM) for 30 min, and later stimulated with LPS (1 μg/ml) for 8 h for expression or 24 h for secretion. Figure 1C shows that the expression of IL-8 is dependent on TLR4, since the treatment with TAK-242 decreased the levels of IL-8 mRNA induced with LPS, whereas Figure 1D shows that TAK-242 prevented IL-8 secretion induced by LPS in CFs. Collectively, LPS regulates the expression and secretion of IL-8 in CFs through TLR4 activation.

### 3.2 Effect of LPS on ICAM-1 and VCAM-1 expression induced by LPS in cardiac fibroblasts

**FIGURE 2:**
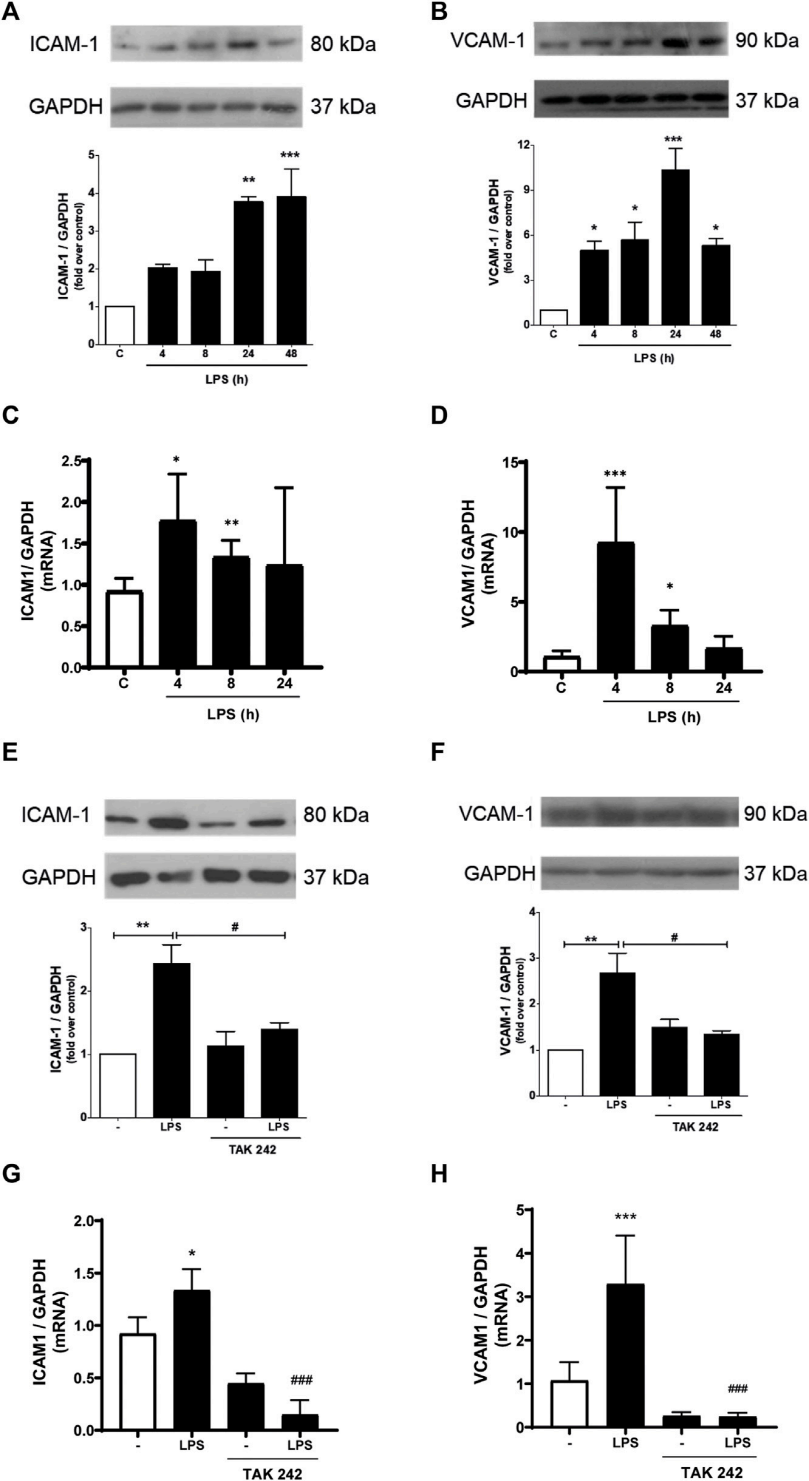
LPS induces ICAM-1 and VCAM-1 expression in cardiac fibroblasts (CF) thorough TLR4. **(A, B)** CF were stimulated with LPS (1 μg/ml) for 4, 8, 24 and 48 h, and then the expression of ICAM-1 **(A)** and VCAM-1 **(B)** was analyzed by WB. A representative image of the experiment is shown in the upper panel and its quantification, in the lower panel. **(C, D)** CF were stimulated with LPS (1 μg/ml) for 4, 8, and 24, and then the mRNA expression of ICAM-1 **(C)** and VCAM-1 **(D)** was analyzed by RT-qPCR. **(E, F)** CF were pre-stimulated with the TLR4 inhibitor TAK-242 (4 uM) for 1 h and subsequently stimulated with LPS (1 μg/ml) for 24 h for the subsequent analysis of ICAM-1 **(C)** and VCAM-1 **(D)** protein expression by WB. A representative image of the experiment is shown in the upper panel and its quantification, in the lower panel Proteins were analyzed by Western Blot. GAPDH was used as a loading control. **(G)** and **(H)** CF were pre-stimulated with the TLR4 inhibitor TAK-242 (4 uM) for 1 h and subsequently stimulated with LPS (1 μg/ml) for 8 h for the subsequent analysis of ICAM-1 **(C)** and VCAM-1 **(D)** mRNA expression by RT-qPCR. ***p < 0.001; **p < 0.01; *p < 0.05 vs. control; #*p* < 0.05 vs. LPS. Results are expressed as mean ± SEM. (*n* = 3).

We subsequently studied whether LPS induced the expression of ICAM-1 and VCAM-1. As shown in Figures 2A, B, CF express ICAM-1 and VCAM-1 proteins. After stimulation with LPS (1 μg/ml) ICAM-1 and VCAM-1 protein levels increased, reaching a maximum expression at 24 h and being statistically significant from 4 h for VCAM-1 and 24 h for ICAM-1 after incubation with LPS. To complement these results, we evaluated the mRNA of ICAM-1 and VCAM-1. Figures 2C, D show that LPS (1 μg/ml) increase both ICAM-1 and VCAM-1 mRNA. Then we proceeded to determine if ICAM-1 and VCAM-1 expression induced by LPS was dependent on the activation of TLR4. CF were pre-stimulated with a TLR4 inhibitor, TAK-242 (4 μM) for 30 min, and later stimulated with LPS (1 μg/ml) for 8 h for mRNA analysis and 24 h for protein analysis. Figures 2E, F show that ICAM-1 and VCAM-1 protein expression is TLR4-dependent, since TAK-242 prevents the LPS-induced increase in ICAM-1 and VCAM-1 protein level, whereas Figures 2G, H show that TLR4 inhibition with TAK-242 prevent LPS effects on ICAM-1 and VCAM-1 mRNA.

### 3.3 Effect of the IFN-β/JAK pathway on IL-8, ICAM-1 and VCAM-1 expression levels induced by LPS

**FIGURE 3:**
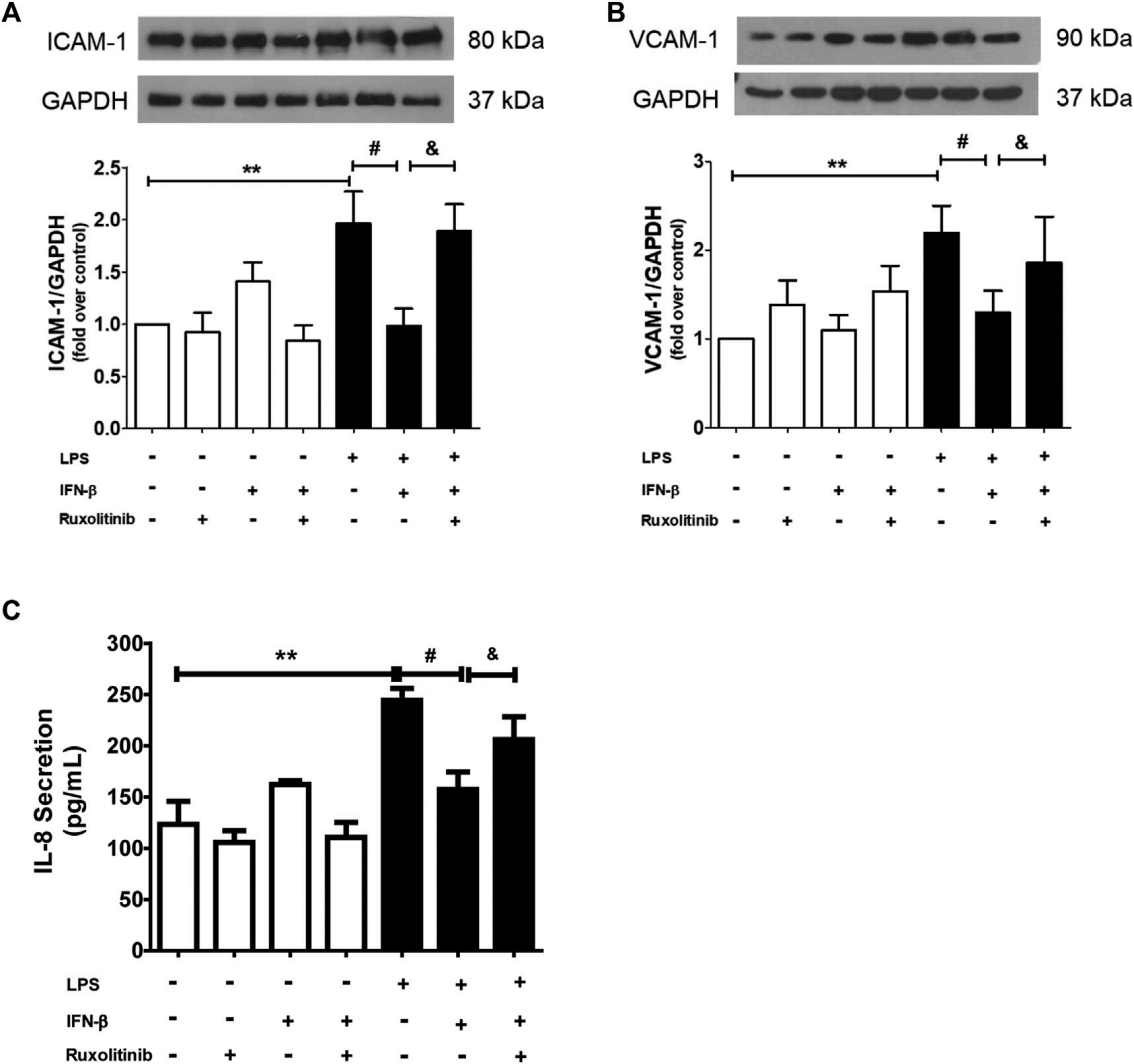
Effect of the IFN/JAK pathway on IL-8 secretion and ICAM and VCAM expression in cardiac fibroblasts (CF). The CF were pre-stimulated with ruxolitinib 30 min before treatment with IFN-β; then CF were pre-stimulated with IFN-β (500 IU) for 1 h and subsequently stimulated with LPS (1 μg/ml) for 24 h. **(A)** The expression of ICAM-1 and VCAM-1 protein levels were analyzed by Western Blot. GAPDH was used as a loading control. A representative image of the experiment is shown in the upper panel and its quantification, in **(C)**. **(B)** IL-8 secretion was analyzed by ELISA from cell culture media. **p < 0.01 vs. control; #*p* < 0.01 vs. LPS; &*p* < 0.05 vs. LPS + IFN-β. Results are expressed as mean ± SEM. (*n* = 3).

Data from our laboratory has shown that IFN-β exerts anti-inflammatory effects on CF when they are stimulated with LPS, and that these effects are mediated by activation of STAT proteins. To demonstrate the role of JAK in the anti-inflammatory effects of IFN-β on CF, the CF were stimulated with ruxolitinib (500 nM), a selective JAK inhibitor, for 30 min. Then CF were stimulated with IFN-β (500 UI/mL) for 1 h; and later with LPS for 24 h to induce inflammation markers on CF. Figures 3A, B show that IFN-β prevents the increase in ICAM-1 and VCAM-1 protein expression levels induced by LPS. Similar results are showed in Figure 3C, where IFN-β prevented the increase in IL-8 secretion induced by LPS. JAK inhibition by ruxolitinib blocked the anti-inflammatory effects induced by IFN-β in CF incubated with LPS.

### 3.4 Effect of the IFN-β/JAK signaling pathway on neutrophil migration

**FIGURE 4:**
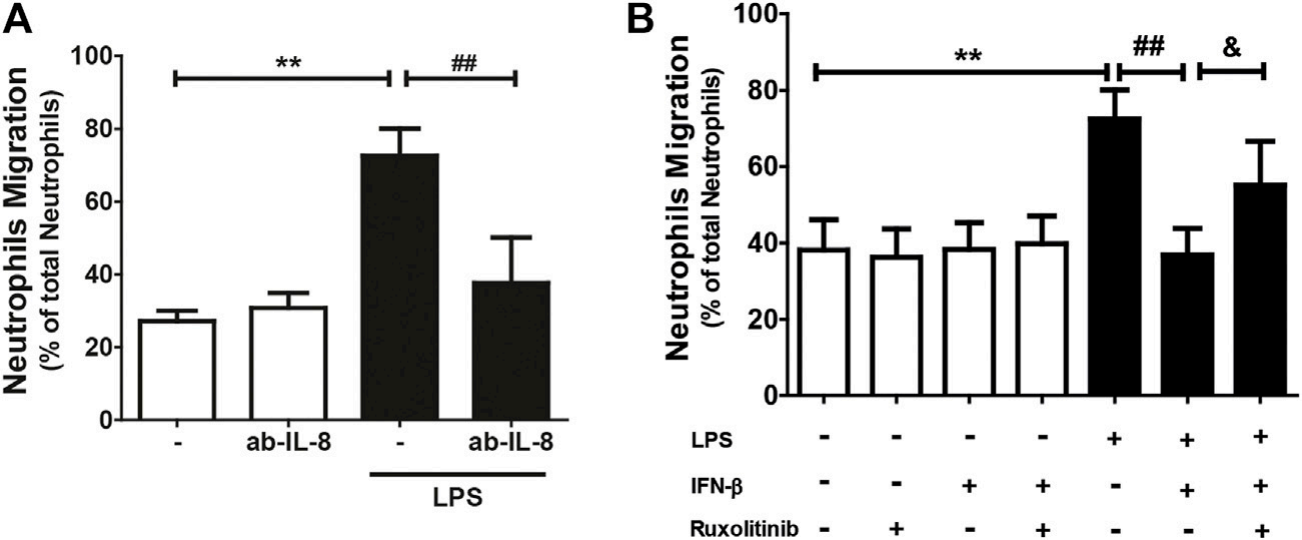
The IFN-β/JAK signaling pathway prevents LPS-induced IL-8-dependent neutrophil migration. Neutrophils were previously labeled with Calcein^(R)^, a fluorescent probe, and seeded in a Transwell plate. The migration was analyzed at 3 h. **(A)** Migration of neutrophils in conditioned culture medium secreted by CF treated with/without LPS (1 μg/ml) for 24 h. The culture medium was preincubated for 30 min with the anti-IL-8 blocking antibody. **(B)** Migration of neutrophils in conditioned culture medium secreted by CF treated with ruxolitinib for 30 min before treatment with IFN-β. Then the cells were stimulated with IFN-β (500 IU/mL) for 1 h, and later stimulated with/without LPS (1 μg/ml) for 24 h. The number of fluorescent neutrophils that migrated to the lower compartment was measured by a spectrofluorometer. The percentage of migration was expressed based on the measured fluorescence vs. the fluorescence emitted by the total aggregated neutrophils. Results represent the mean ± SEM of four independent experiments. **p < 0.01 vs. control; #*p* < 0.01; ##*p* < 0.01 vs. LPS and and*p* < 0.05 vs. LPS + IFN-β. Results are expressed as mean ± SEM. (*n* = 3).

Since IL-8 is a chemoattractant for neutrophils, and since CF secrete this chemokine and express the ICAM-1 and VCAM-1 proteins, we proceeded to isolate and purify neutrophils to perform neutrophil recruitment studies (migration and adhesion) on the CF. Neutrophil migration was stimulated by conditioned culture media of CF treated with: LPS, IFN-β, IFN-β + LPS, and ruxolitinib + IFN-β + LPS. The results in Figure 4A show that the culture medium secreted by LPS-treated CF increased neutrophil migration compared to the conditioned medium obtained from untreated CF (25% *versus* 70%). This effect was blocked by the action of an anti-IL-8 antibody, which, by itself, did not modify basal migration. As we previously showed that JAK inhibition prevented the anti-inflammatory effects of IFN-β on LPS-induced IL-8 expression in CF, our next step was to determine whether ruxolitinib affected neutrophil migration. Figure 4B shows that the conditioned medium of CF incubated with IFN-β reduced the migration of neutrophils induced by the conditioned medium of CF treated only with LPS (45% vs. 70%), while the conditioned medium of CF incubated with ruxolitinib inhibited the antimigratory capacity observed with IFN-β (60% vs. 45%). These data agree with the results observed in the secretion of IL-8. Taken together, these results suggest that LPS increases IL-8 secretion and thus, neutrophil migration. IFN-β reduces pro-inflammatory effects of LPS, while JAK is necessary for the anti-inflammatory effects induced by IFN-β.

### 3.5 Effect of IFN-β/JAK on neutrophil adhesion in a cardiac fibroblast monolayer

**FIGURE 5:**
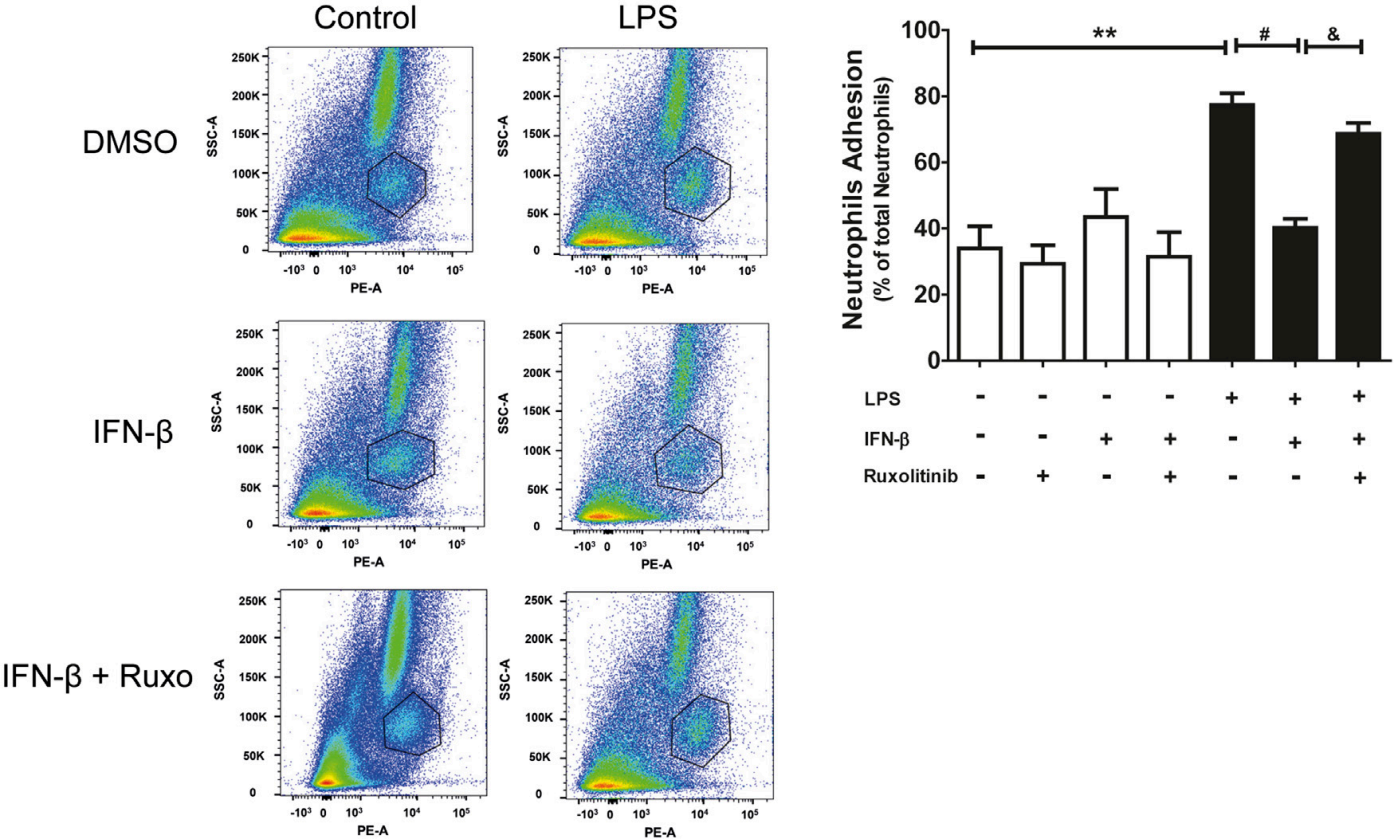
The IFN-β/JAK signaling pathway prevents LPS-induced neutrophil adhesion. Representative diagrams of adhesion of neutrophils to CF pre-stimulated with IFN-β (500 IU/mL) for 1 h and/or LPS (LPS (1 μg/ml) for 24 h in the absence of ruxolitinib (**(A)**: cardiac fibroblast; **(B)** neutrophils and **(C)** other leukocytes and cell death). CF with adherent leukocytes were trypsinized for their removal from the plate and then were incubated with the anti RP-1(PE) antibody, specific for neutrophils, for 30 min. Adhesion levels were determined by flow cytometry. The graph shows adhesion levels of neutrophils to a CF monolayer treated as observed in the graph. The percentage of neutrophil adhesion was calculated as follows: 100 x (n° cells RP-1^+^ adhered/n° total cells RP-1^+^). **p < 0.01 vs. control; ##*p* < 0.01 vs. LPS; &&*p* < 0.01 vs. LPS + IFN-β. Results are expressed as mean ± SEM (*n* = 3).

Since CF express the ICAM-1 and VCAM-1 proteins, neutrophil adhesion assays were carried out on a CF monolayer. Adhesion was determined by using the specific anti-RP-1 antibody against neutrophils, and subsequent analysis by Flow Cytometry. Figure 5 shows representative diagrams obtained by flow cytometry indicating the percentage of the population of neutrophils and CF detected (a: cardiac fibroblast; b: neutrophils and c: other leukocytes and death cells). In addition, Figure 5 shows the quantification of the histograms we obtained, observing that IFN-β reduced the adhesion of neutrophils to the LPS-induced CF monolayer (70% vs. 40%), which correlates with the data related to ICAM-1 and VCAM-1 expression levels. On the other hand, JAK inhibition with ruxolitinib prevented the anti-inflammatory effect of IFN-β; we observed greater adhesion of neutrophils to the CF monolayer in cells treated with ruxolitinib + IFN-β + LPS (70%), *versus* those treated with IFN-β + LPS (30%). Taken together, our data suggest that LPS increases neutrophil adhesion on CF and that IFN-β reduces the LPS pro-inflammatory effects, while JAK is required for IFN-β-induced anti-inflammatory effects.

### 3.6 Effect of IFN-β/JAK on metalloproteases activity from a treated cardiac fibroblast monolayer and cardiac fibroblast/neutrophils interaction

**FIGURE 6:**
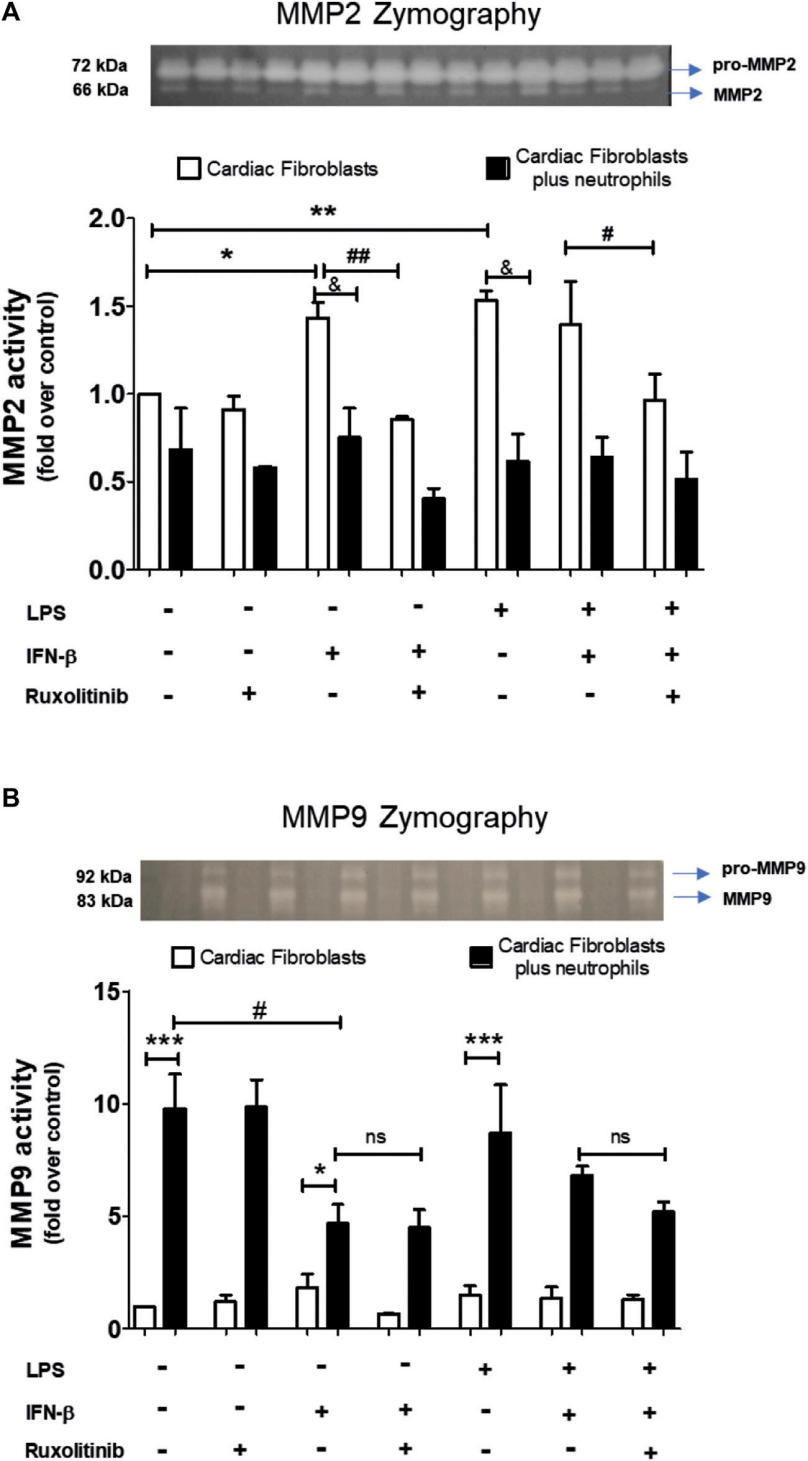
Activity of MMP2 and MMP9 in cardiac fibroblasts and CF-neutrophils co-cultures. **(A)** CF were pre-stimulated with ruxolitinib for 30 min before IFN-β treatment. CF were pre-stimulated with IFN-beta (500 IU/mL) for 1 h and subsequently stimulated with LPS (1 μg/ml) for 24 h. In treated CF alone and in pre-treated CF co-cultured with the MMPs activity was measured as indicated in materials and methods. A representative image of the experiment is shown in the upper panel and its quantification appears in the lower panel. **(A)** MMP2 activity in CF alone (white bars) and in a CF-Neutrophils co-culture (black bars). *p < 0.05; **p < 0.01 vs. non-treated CF; #*p* < 0.05 Ruxolitinib + IFN-β vs. Ruxolitinib + IFN-β + LPS; ##*p* < 0.01 IFN-β vs. Ruxolitinib + IFN-β; &*p* < 0.05 CF vs. CF + neutrophils. **(B)** MMP9 activity in CF alone (white bars) and in a co-culture of CF-Neutrophils (black bars).). *p < 0.05; ***p < 0.001 CF vs. treated CF-neutrophils; #*p* < 0.05 no treated CF vs. IFN-β and Ruxolitinib + IFN-β. Results are expressed as mean ± SEM. (*n* = 3).

A common feature of CF and neutrophils is their ability to secrete matrix MMPs. However, the consequences of the effect of the interaction between CF and neutrophils on the secretion of MMPs is unknown. Therefore, we performed zymography studies to assess the activity of MMPs. Zymography in gelatin gels of the culture medium of treated-CF alone or treated-CF co-cultured with neutrophils showed specific bands corresponding to the molecular weights of MMP-2 and MMP-9 (66 and 83 kDa, respectively; Figures 6A, B). Figure 6A shows that LPS, IFN-β, LPS + IFN-β increased MMP2 activity in CF alone respect unstimulated CF (noted as white bars* in the graph). JAK inhibition with ruxolitinib prevented the effect of IFN-β on MMP2 activity, both alone and in the presence of LPS (indicated as white bars# on the graph). In the co-culture conditions between treated-CF and neutrophils (black bars), the presence of neutrophils did not affect the MMP2 activity of CF in the basal state, while the co-culture reduced the effects produced by IFN-β, LPS and LPS + IFN on CF respect MMP2 activity (indicated as black barsand in the graph).

In Figure 6B, we show that in CF alone the MMP9 activity was not affected by any of the stimuli used (white bars); however, MMP9 activity increased in the co-culture between treated-CF and neutrophils in all conditions (black bars) (noted as * in the graph). LPS did not affect the activity of MMP9 in any of the evaluated conditions, being like unstimulated CF. In the IFN-β-treated CF, the co-culture between CF and neutrophils a reduced MMP9 activity respect unstimulated CF was observed (denoted as black bars# in the graph), and these effects was not modified by JAK inhibition with ruxolitinib.

## 4 Discussion

The main results of this study show that LPS increases IL-8 mRNA expression and IL-8 protein secretion, and ICAM-1 and VCAM-1 mRNA and protein expression in CF. These effects were inhibited by the IFN-β/JAK signaling pathway. IL-8, ICAM-1, and VCAM-1 are required for neutrophil recruitment (migration and adhesion, respectively). MMP2 activity was not greatly affected by a co-culture between CF and neutrophils; however, LPS and IFN-β induced MMP2 secretion and activity in CF. MMP9 activity was increased in the co-culture between CF and neutrophils, being modulated by treatment with IFN-β, but not with LPS.

### 4.1 Interleukin-8 expression in LPS-stimulated CF

Our results show that CF has IL-8 basal expression level, and that LPS stimulates an early increase in its mRNA expression and protein secretion. In this regard, previous reports have indicated that LPS increases the expression levels of IL-8 in human CF (Skioldebrand et al., 2017; Li et al., 2021), and in fibroblasts derived from nasal polyps, periodontal ligament, and lung polyps, among others (Zhang et al., 2011; Cho et al., 2014; Zhang and Li, 2015). Increased levels of IL-8 secretion have also been described in small intestine fibroblasts, although at very long periods after stimulation (24–72 h) (Chakravortty and Kumar, 1999). To date, an increase in IL-8 plasmatic levels has been documented after a heart attack (de Winter et al., 1997). The results of our study show that there is an increase in the secretion of IL-8 after 8 h of stimulation with LPS, results that coincide with the decrease in its intracellular mRNA expression levels. IL-8 secretion reaches a maximum level after 24 h of stimulation, decreasing at later times. These results coincide with those described in the literature regarding the stimulus time in which the greatest amount of protein is secreted (Chakravortty and Kumar, 1999; Wang et al., 2011). Our results showed that LPS-induced IL-8 secretion is dependent on TLR4. Similar results have been reported in other studies, which have indicated the participation of TLR4 in the expression of IL-8 and the participation of the MAPK pathway in a signaling pathway that leads to the IL-8 secretion (Cho et al., 2014). The participation of the MyD88 protein in IL-8 secretion in lung fibroblasts has also been observed (Smith et al., 2001). Although we have no results showing that NF-kB inhibition prevents IL-8 secretion, data from our laboratory show that LPS activates AKT, ERK1/2 and NF-kB (Boza et al., 2016); and it is well documented in the literature that NF-KB regulates the expression of IL-8; although in this case it was because of IL-1beta (Mizuno et al., 2022). Mukaida et al. (Mukaida et al., 1994), reported that the transcription of the IL-8 gene requires the activation of a series of transcription factors, including NF-kB. Sakuta et al. (Sakuta et al., 1998), confirmed this fact by treating gingival fibroblasts with an NF-kB inhibitor, which decreased IL-8 gene expression. These precedents suggest the participation of both NF-kβ and AP-1 at the transcriptional level for the synthesis, and secretion of IL-8, via the MyD88-dependent TLR4 pathway. In this regard, previous results from our laboratory have shown that LPS induces the activation of NF-kβ (Boza et al., 2016); therefore, these results suggest that in CF these signaling pathways would be the activated inducing expression and release of IL-8.

Regarding ICAM-1 and VCAM-1 expression induced by LPS, it is well known that the expression of adhesion molecules by cardiac cells is a vital event for inflammatory processes. High levels of these proteins have been found in various cardiac inflammatory processes, such as ischemia/reperfusion and myocarditis (Kukielka et al., 1993). In this context, elevated expression of ICAM-1 and VCAM-1 has been associated with leukocyte mobilization and infiltration into cardiac tissue. These proteins are constitutively expressed in various cell types, and their expression is induced by proinflammatory stimuli, such as IL-1β, LPS, TNF-α and IFN-γ, among others (Hosokawa et al., 2006; Yang et al., 2010; Chang et al., 2019; Shen et al., 2021). Our current results confirm previous findings obtained in our laboratory, in which we have shown that TLR4 activation in CF can increase the mRNA and protein expression levels of ICAM-1 and VCAM-1, obtaining a maximum after 24 h of stimulation with LPS. Taken together, these results highlight the proinflammatory effects of LPS in CF (Humeres et al., 2016; Bolivar et al., 2018).

### 4.2 Effect of IFN-β on the secretion of IL-8 and ICAM-1 and VCAM-1 expression in cardiac fibroblasts

IFN-β elicit a wide variety of anti-inflammatory, pro-inflammatory, antiviral, and antibacterial responses, and can regulate the development and activation of most effector cells of the innate and adaptive immune responses. (Darnell et al., 1994; Ihle, 1995; Chen et al., 2004; Pestka et al., 2004; Noppert et al., 2007; Zurney et al., 2007; Ivashkiv and Donlin, 2014). IFN-β can be produced by various cells of the innate immune system (Applequist et al., 2002; Takeda and Akira, 2004), as well as by fibroblasts and epithelial cells (Ivashkiv and Donlin, 2014). Due to the description of the anti-inflammatory capacity of IFN-β, its expression via TLR4 and the presence of this receptor in CF, we decided to study its role as a mediator in the inflammatory process that involves the activation and recruitment of neutrophils. The levels of IL-8 induced by LPS were completely reduced in pre-treated CF with IFN-β, reaching values like the controls. In this regard, Oliveira et al. (Oliveira et al., 1992), described that IFN-β inhibits the transcription of IL-8 mRNA induced by TNF-α in skin fibroblasts. This effect was attributed to IFN-β induced proteins, which act by inhibiting the transcription of pro-inflammatory genes (Sen and Lengyel, 1992). A decrease in the accumulation of IL-8 was also detected in skin fibroblast, attributing it to the fact that IFN-β blocks the synthesis of IL-8 at the posttranscriptional, translational, or posttranslational levels. Similar results were found in melanoma cells (Singh et al., 1996). Other authors have already shown that IFN-β inhibits the secretion of IL-8 through a mechanism that involves the Stat1, Stat2 and IRF-9 proteins (Laver et al., 2008). In this regard, in a previous work in our laboratory we have shown that in TLR4-activated CF (pre-activated with LPS, and consequently), IFN-β activates the STAT2 and/or STAT3 proteins, producing an anti-inflammatory effect reducing the secretion of IL-1beta, TNF-alpha, IL-6, MCP-1 and IP10 (Bolivar et al., 2018). These results reinforce our findings, and we could suggest that IFN-β through the activation of STAT2 or STAT3 proteins could reduce the secretion of IL-8. Whereas the anti-inflammatory effects of IFN-β by itself could be mediated by SAT3 activation. We certainly do not rule out that other processes may also be involved. Collectively, all these results obtained in CF establish a relationship between IFN-β, as an anti-inflammatory agent by decreasing the expression of IL-8.

The reduction of ICAM-1 and VCAM-1 proteins by IFN-β follows the same dynamics as the effect produced on IL-8. Previously, our laboratory demonstrated that IFN-β has a dual behavior on CF, since under pro-inflammatory conditions induced by LPS, IFN-β exerts an anti-inflammatory effect; while in non-inflammatory conditions, it exerts a pro-inflammatory effect (Bolivar et al., 2018). In this regard, Floris et al., described that ICAM-1 and VCAM-1 expression levels decreased in a rat model of multiple sclerosis treated with IFN-β (Floris et al., 2002). However, these results differ with those reported by Dhib-Jalbut et al., (Dhib-Jalbut et al., 1996), who described that the effect of IFN-β on the expression of the ICAM-1 and VCAM-1 induced by TNF- α, IL-1β or IFN-γ was additive in umbilical cord endothelial cells and associated with a slight increase in lymphocyte adhesion to endothelial cells. Our results showed that IFN-β effects were mediated by JAK signaling pathway-dependent activation, since IFN-β effects were inhibited by ruxolitinib. In this regard, previously we have showed that ruxolitinib does not modulate the effects of LPS in fibroblasts in terms of cytokine secretion (IL-6, TNF-alpha, MCP-1, IL-10, and IP-10) [27. Consequently, the effect of LPS in the presence of roxilitinib was not evaluated, due to the aforementioned background.

### 4.3 Effect of IFN-β on the migration and adhesion of neutrophils to cardiac fibroblasts

Neutrophils are recognized as one of the most important participants in the body’s defense and inflammatory process (Morzycki and Issekutz, 1991; Dhib-Jalbut et al., 1996). Our results showed an evident increase in the percentage of adhesion of neutrophils on CF stimulated with LPS. The migration of neutrophils through the endothelium is important and necessary to reach the site of inflammation and once there, to degranulate and release their content rich in cytokines, chemokines, and enzymes, including MMPs (Wright et al., 2010). Neutrophil adhesion to lung and heart fibroblasts has been documented (Morzycki and Issekutz, 1991; Gao and Issekutz, 1996; Shang and Issekutz, 1997; Couture et al., 2009). Moreover, a report described a preventive effect exerted by IFN-β on the infiltration of neutrophils induced by IL-8 in the rat brain (Veldhuis et al., 2003). In the present study, IFN-β prevented migration and adhesion of neutrophils to CF treated with LPS. In addition, and as mentioned above the recruitment of neutrophils to the site of injury is stimulated through chemotaxis exerted by IL-8, which also plays a role as a neutrophil activator. IL-8 triggers an increase in the expression glycolipids and integrins on neutrophil surface, thus increasing the expression of proteins that allow interaction with ICAM-1 or VCAM-1 proteins expressed in CF, or other cell types (Wright et al., 2010). For this reason, the effect produced by IFN-β on the adhesion of neutrophils to CF stimulated with LPS, is consistent with the preventive effect that, in the same way, is produced on the levels of IL-8 and ICAM-1 under the same conditions. Thus, part of the novelty of this work lies in the secretion of IL-8 from the CF and in the expression of ICAM-1 and VCAM-1 on its surface, which enables the recruitment of neutrophils, which is regulated by IFN-β.

### 4.4 Effect of IFN-β on the secretion of MMP2 and MMP9

MMPs denature and degrade fibrillar collagens and other components of the ECM. Several studies have shown that the deregulation of MMPs is involved in the development of myocardial extracellular matrix remodeling and cardiac fibrosis (Fan and Kassiri, 2021). The interaction between CF and cells of the immune system has been shown to have important pathophysiological consequences (Okyere and Tilley, 2020). We previously demonstrated that the interaction between CF and monocytes favors a differentiation towards a M1 or M2 macrophage phenotype, which depends on the pre-stimulation of the CF and the cytokines secreted by the CF because of this stimulus (Humeres et al., 2016). The interaction between CF and macrophages favors the secretion of TGF-β, a growth factor that induces the differentiation of CF in to cardiac myofibroblast**,** and a greater secretion of collagens, favoring the tissue repair process (Hitscherich and Lee, 2021).

This study provides new insights into the interaction between CF and neutrophils, and in particular, regarding the activity of MMPs. Our results showed that in the co-culture between CF and neutrophils, MMP2 activity arises mainly from the CF, while MMP9 activity could be originates mainly from neutrophils. In cardiac tissue, the expression of MMP2 and MMP9 has been related to both the migration of immune system cells and part of the inflammatory response; as well as to the infiltration of CF to the site of the damage to proceed with the repair of the damaged tissue (Brown et al., 2007). Our results regarding the increase in MMP2 activity by LPS and IFN-β in CF were like those activated by IL-1β, although this cytokine induces a greater spectrum of MMPs that includes MMP-2, 3 and 9; moreover, our results are also consistent with previous reports in CF (Siwik et al., 2000; Meiners et al., 2004; Xie et al., 2004). On the other hand, the release and activity of MMP9 has been described as a consequence of neutrophil activation. In fact, the activation of neutrophils leads to their degranulation, releasing a complex set of proteins, cytokines and MMPs, among which MMP8 and MMP9 stand out (Faurschou and Borregaard, 2003; Zhang et al., 2022). Our results demonstrate that the interaction between CF and co-cultured neutrophils is a sufficient stimulus to trigger neutrophil activation, leading to increased MMP9 release. Similar to the results we observed for IL-8, ICAM-1, and VCAM-1, the effect of LPS on MMP2 activity was inhibited by IFN-β through activation of the JAK pathway. The effects of IFN-β showed a reduction in MMP9 activity, which would coincide with the anti-inflammatory effects that we have described for IFN-β; however, this effect was not inhibited by ruxolitinib. The cause of this last result is unknown, and it is contrary to our previous results; therefore, it needs a deeper evaluation that might explain this difference.

Indeed, our results show that MMP2 comes from the CF. This result is consistent with what has been described in the literature (Turner and Porter, 2012). Furthermore, pro-inflammatory stimuli induce the release of MMP2. In this sense, we have previously shown that IFN-β behaves as a pro-inflammatory (when there is no prior inflammatory process), inducing the activity of MMPs (as observed in this results). However, when there is a pro-inflammatory process, IFN-β behaves as an anti-inflammatory (Bolivar et al., 2018). Although in our results was observed that the activity of MMPs in the presence of LPS + IFN did not decrease. This suggests that other mechanisms could be involved.

Taken together, our results suggest that in CF alone LPS, IFN-β, IFN-β + LPS increases MMP2 activity; while the co-culture of treated-CF with neutrophils, a reduction in MMP2 activity was observed. The co-culture of treated ad non-treated-CF with neutrophils increases the activity of MMP9; although in IFN-β-treated CF co-cultured with neutrophils MMP9 activity was lower than unstimulated CF. The effects of IFN-β did not require the participation of JAK. These data suggest that activation of MMP species plays a permissive role in releasing ECM constraints on cell motility in fibroblast-directed migration.

## 5 Conclusion

In the present study we show that LPS induces a proinflammatory response, which leads to the recruitment of neutrophils, which is counteracted by the IFN-β/JAK signaling pathway. The pretreatment of CF with IFN-β, prior to the stimulation with LPS, triggers a decrease in IL-8 levels, as well as a decrease in ICAM-1 and VCAM-1 expression levels, while such anti-inflammatory effects are blocked by JAK inhibition. Finally, we show that IFN-β decreases the recruitment of neutrophils by CF. Because of this recruitment, the release of MMP9 by neutrophils is induced. These results highlight the potent participation of CF in inflammatory responses, and in stimulating the recruitment of neutrophils.

## Data Availability

The raw data supporting the conclusions of this article will be made available by the authors, without undue reservation.
