# Supplementary figures and images for: Interferon-β decreases LPS-induced neutrophil recruitment to cardiac fibroblasts
Source: Front Cell Dev Biol. 2023 Sep 20;11:1122408. doi: 10.3389/fcell.2023.1122408 (PMC10547890; doi:10.3389/fcell.2023.1122408)

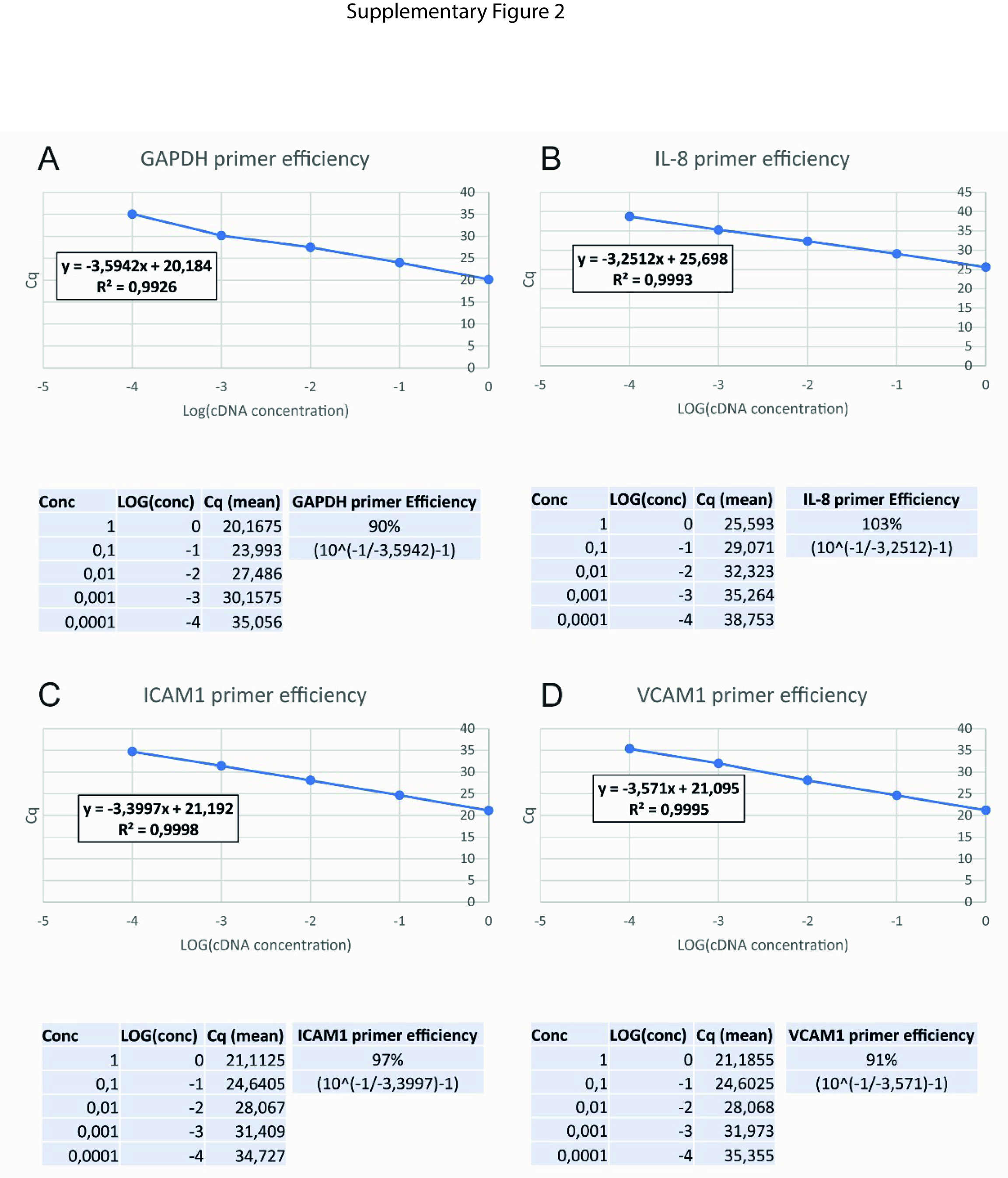

Supplement: Supplementary file 3 [file Image3.TIF]

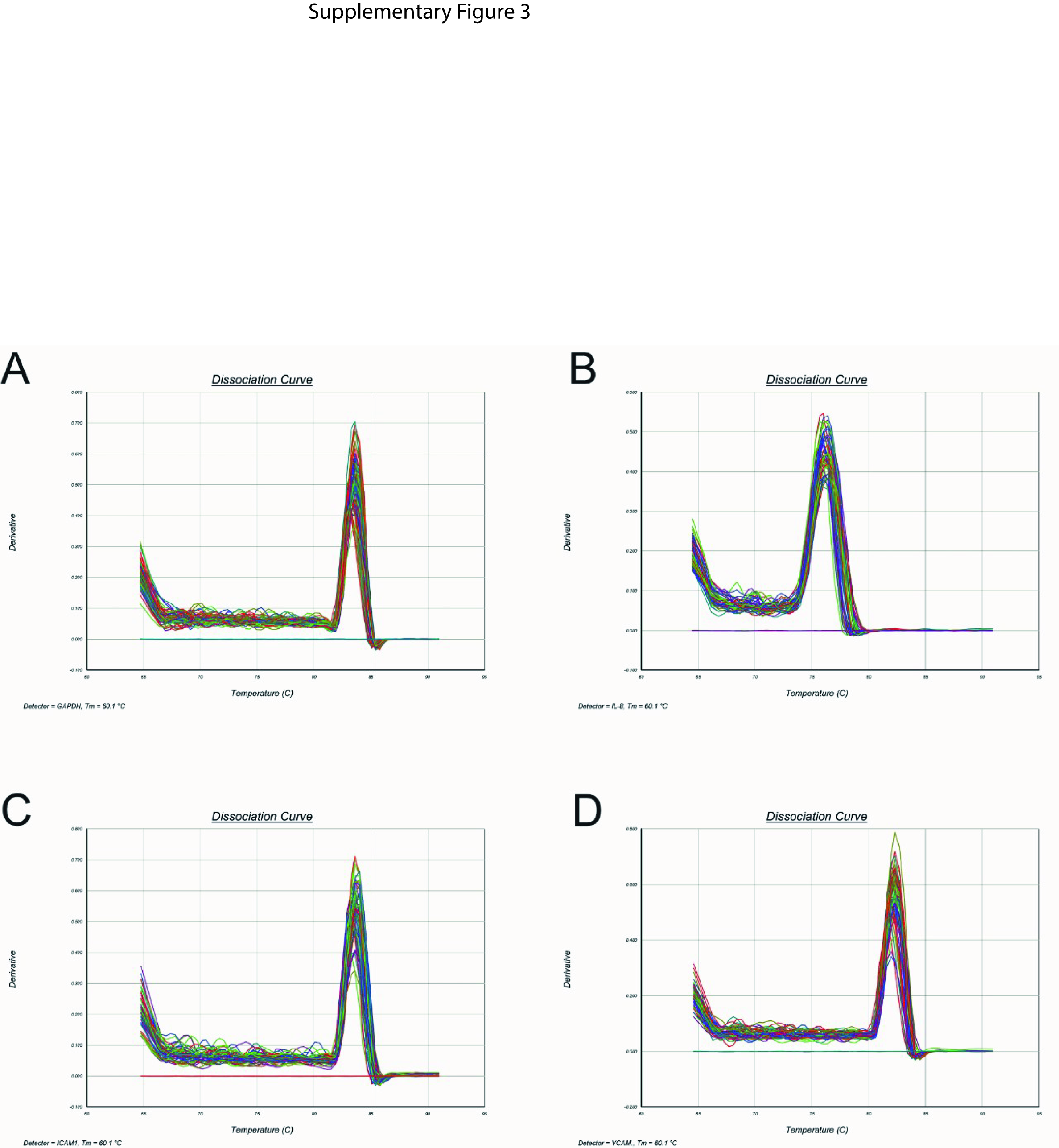

Supplement: Supplementary file 4 [file Image4.TIF]

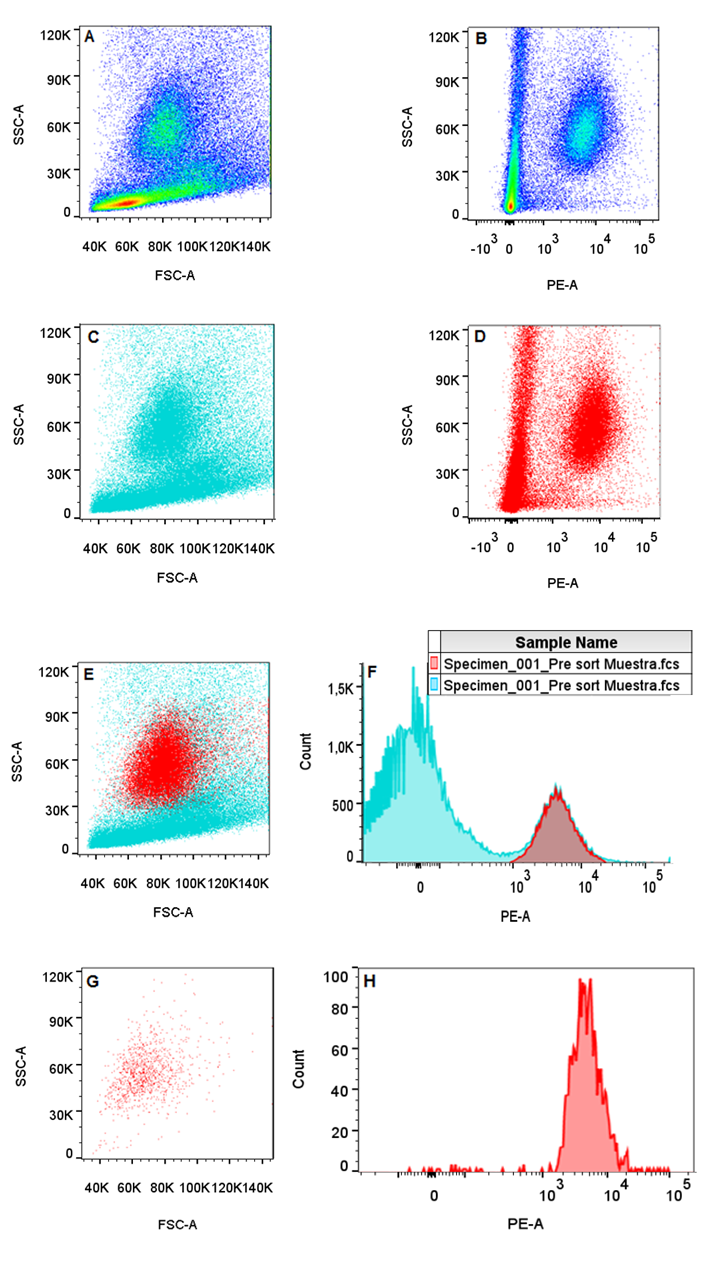

Supplement: Supplementary file 5 [file Image2.TIF]

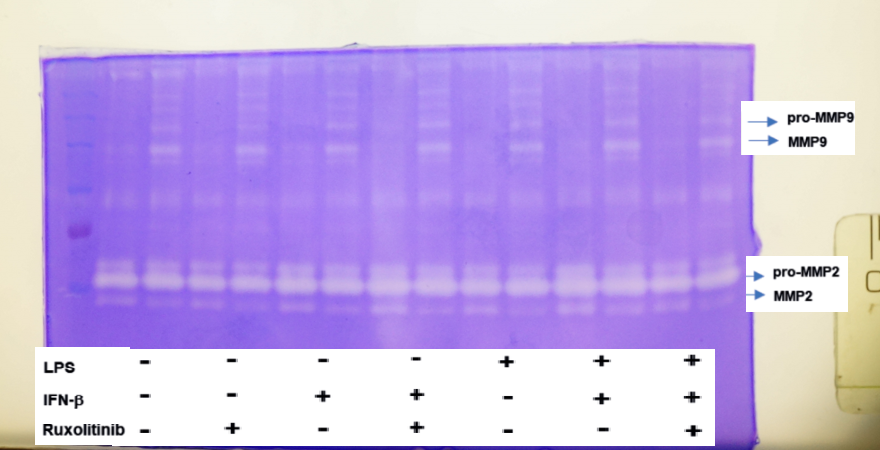

Supplement: Supplementary file 7 [file Image1.PNG]
